# Targeting the gut to prevent sepsis from a cutaneous burn

**DOI:** 10.1172/jci.insight.137128

**Published:** 2020-10-02

**Authors:** Fatemeh Adiliaghdam, Paul Cavallaro, Vidisha Mohad, Marianna Almpani, Florian Kühn, Mohammad Hadi Gharedaghi, Mehran Najibi, Laurence G. Rahme, Richard A. Hodin

**Affiliations:** 1 Department of Surgery, Massachusetts General Hospital, Harvard Medical School, Boston, Massachusetts, USA.; 2 Shriners Hospital for Children, Boston, Massachusetts, USA.; 3 Department of Microbiology and Immunobiology, Harvard Medical School, Boston, Massachusetts, USA.; 4 Department of General, Visceral and Transplant Surgery, Hospital of the University of Munich, Munich, Germany.

**Keywords:** Gastroenterology, Microbiology, Bacterial infections, Mouse models

## Abstract

Severe burn injury induces gut barrier dysfunction and subsequently a profound systemic inflammatory response. In the present study, we examined the role of the small intestinal brush border enzyme, intestinal alkaline phosphatase (IAP), in preserving gut barrier function and preventing systemic inflammation after burn wound infection in mice. Mice were subjected to a 30% total body surface area dorsal burn with or without intradermal injection of *Pseudomonas aeruginosa*. Mice were gavaged with 2000 units of IAP or vehicle at 3 and 12 hours after the insult. We found that both endogenously produced and exogenously supplemented IAP significantly reduced gut barrier damage, decreased bacterial translocation to the systemic organs, attenuated systemic inflammation, and improved survival in this burn wound infection model. IAP attenuated liver inflammation and reduced the proinflammatory characteristics of portal serum. Furthermore, we found that intestinal luminal contents of burn wound–infected mice negatively impacted the intestinal epithelial integrity compared with luminal contents of control mice and that IAP supplementation preserved monolayer integrity. These results indicate that oral IAP therapy may represent an approach to preserving gut barrier function, blocking proinflammatory triggers from entering the portal system, preventing gut-induced systemic inflammation, and improving survival after severe burn injuries.

## Abstract 2

Oral intestinal alkaline phosphatase therapy preserves gut barrier function in mice following burn injury, blocking pro-inflammatory triggers from entering the portal system, preventing gut-induced systemic inflammation, and improving survival.

## Graphical abstract

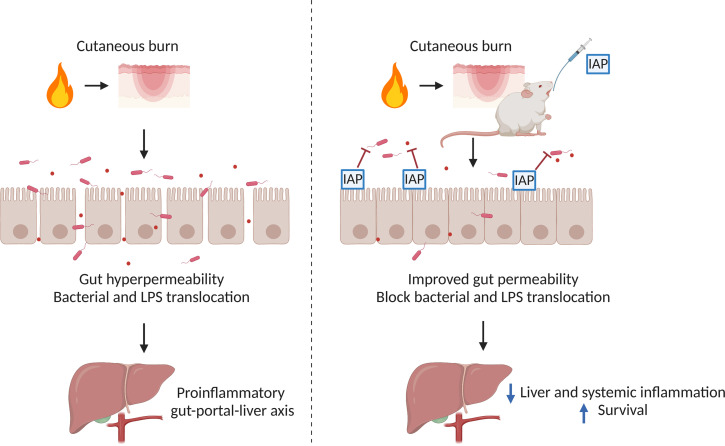

## Introduction

Patients with severe cutaneous burn injury are at risk for developing systemic sepsis, in some cases leading to shock, multiorgan failure (MOF), and even death (1). In fact, thermal injury is one of the leading causes of mortality and morbidity worldwide, causing over 300,000 deaths per year, and it has been estimated that 75% of all deaths are related to sepsis or infectious complications. Although the precise source of this systemic inflammation and sepsis remains unclear, it has been suggested that the gut may serve as a portal of entry for intraluminal bacteria and proinflammatory triggers, such as LPSs, becoming the main source of systemic inflammation and MOF in critically ill patients (1, 2).

Several mechanisms linking the effects of external burn injury to intestinal barrier dysfunction have been suggested. For example, burn-induced hypovolemia can cause systemic ischemia and hypoperfusion, which may lead to breakdown of the intestinal epithelial layer and tight junctions, impairing barrier integrity and allowing for translocation of bacteria and bacterial-derived mediators. Additionally, a myriad of other elements, such as burn-induced hypoxia, enterocyte apoptosis, and gut microbiome dysbiosis, have been suggested to contribute to intestinal barrier dysfunction after burn injury. Under conditions of intestinal barrier injury and the associated local inflammatory response, bacterial and other pathogenic inflammatory mediators enter the portal venous and mesenteric lymphatic systems, ultimately reaching distant organs. The resultant systemic inflammatory response further injures the gut barrier, establishing a “vicious cycle” of inflammation (2–7). Furthermore, burn wound infection is a frequent complication of burn injury, compounding the initial insult and further affecting the gut barrier (2, 8–11).

Given the apparent importance of an impaired gut barrier in burn-associated morbidity and mortality and infection vulnerability, protecting the intestinal tract after a burn insult may represent a therapeutic approach. Intestinal alkaline phosphatase (IAP), a small intestinal brush border enzyme, is expressed in villus-associated enterocytes and is a critical component of the gut mucosal defense system. IAP is secreted into the intestinal lumen and sits at the interface between the host and the gut microbiota. Importantly, IAP has been shown to protect the host from the potential harmful effects of microbial-derived inflammatory mediators. For example, IAP detoxifies a variety of pathogenic mediators, including LPS and bacterial flagellum, by dephosphorylating active components of these molecules (12–14). We demonstrate that IAP improves tight junction protein (TJP) levels and acts to preserve the intestinal barrier integrity (15). Moreover, this enzyme prevents high-fat diet–induced endotoxemia (16, 17) and maintains gut microbial homeostasis (13, 18–20), and, interestingly, its production is enhanced by enteral nutrition (21), which, in patients with a burn injury, decreases systemic IL-6 and TNF-α and improves burn wound healing (22). Based on these antiinflammatory and barrier-protective functions, we hypothesized that IAP may play a critical role in a clinically relevant murine model of burn wound infection.

## Results

### Lack of IAP resulted in an increased burn site infection–induced gut hyperpermeability and systemic inflammation.

**Figure 1:**
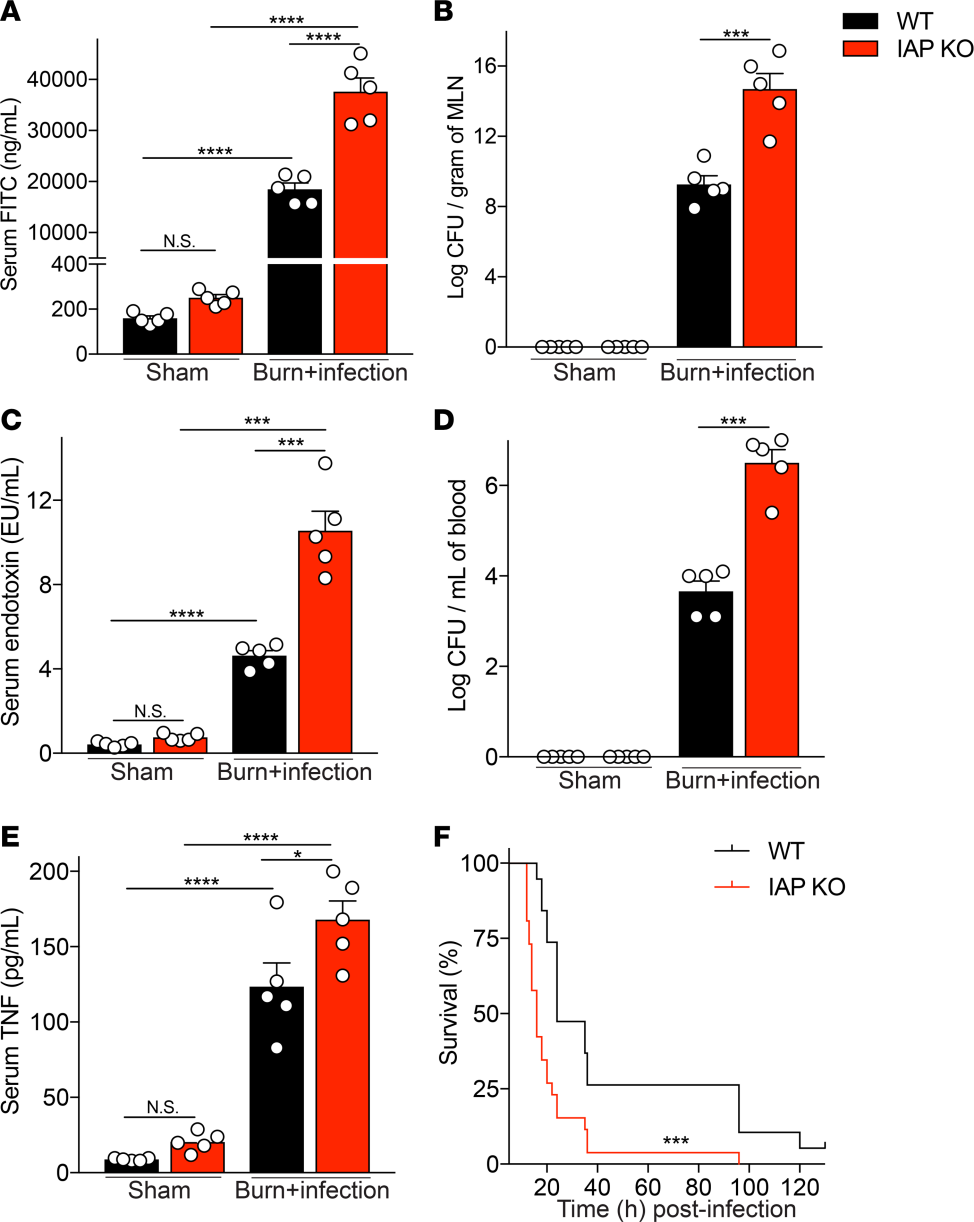
Lack of IAP results in an increased burn site infection–induced gut hyperpermeability, augmented systemic inflammation, and earlier burn site infection–induced death. (**A**) FITC-dextran levels at 18 hours after burn wound infection in serum of WT and IAP-KO mice after 4 hours of intragastric FITC administration. (**B**) Bacterial burden in mesenteric lymph nodes (MLN) of mice expressed as log of colony-forming units (CFU) normalized to tissue weight. (**C**) Serum endotoxin level measured by Limulus amebocyte lysate assay. (**D**) Bacterial burden in systemic blood expressed as log of CFU normalized to blood volume. (**E**) TNF-α levels in serum of WT and IAP-KO mice 18 hours after burn wound infection injury measured by ELISA. (**F**) Survival of WT and IAP-KO mice after receiving burn wound infection insult. For multiple comparisons, 1-way ANOVA with multiple post hoc comparisons using Tukey’s test was performed. Kaplan-Meier survival curve was used for the survival study, and the groups were compared using the log-rank test. Each group included 5 animals and data are representative of 3 biological replicates. The survival study includes 19 WT and 26 *IAP*-KO mice. *P < 0.05, ***P < 0.001, ****P < 0.0001. IAP, intestinal alkaline phosphatase.

Given the role of IAP in detoxifying inflammatory mediators, including LPS in the intestinal lumen and its beneficial effect on gut barrier function, we hypothesized that the mice lacking IAP would display more severe gut barrier damage and systemic inflammation after burn site infection injury. Indeed, we found that IAP-KO mice had significantly higher absorption of orally gavaged FITC-dextran in their blood compared with their WT counterparts, indicating an increased gut permeability after a burn site infection in KO mice (Figure 1A). As expected, the higher gut permeability was associated with an increased bacterial translocation to the mesenteric lymph nodes (MLNs) in the KO compared with the WT mice (Figure 1B). We then explored the systemic impact of greater intestinal hyperpermeability and increased MLN bacterial load. We found that IAP-KO mice had significantly higher systemic LPS concentrations compared with WT mice (Figure 1C). We also observed a higher systemic bacterial load (Figure 1D) and increased systemic inflammation (Figure 1E) in KO mice compared with WT controls. Finally, lack of IAP accelerated burn wound infection–induced death (median survival: 16 vs. 24 hours, KO vs. WT mice, *P* < 0.0001) (Figure 1F).

### IAP supplementation reduced burn site infection–induced gut barrier damage after burn injury.

**Figure 2:**
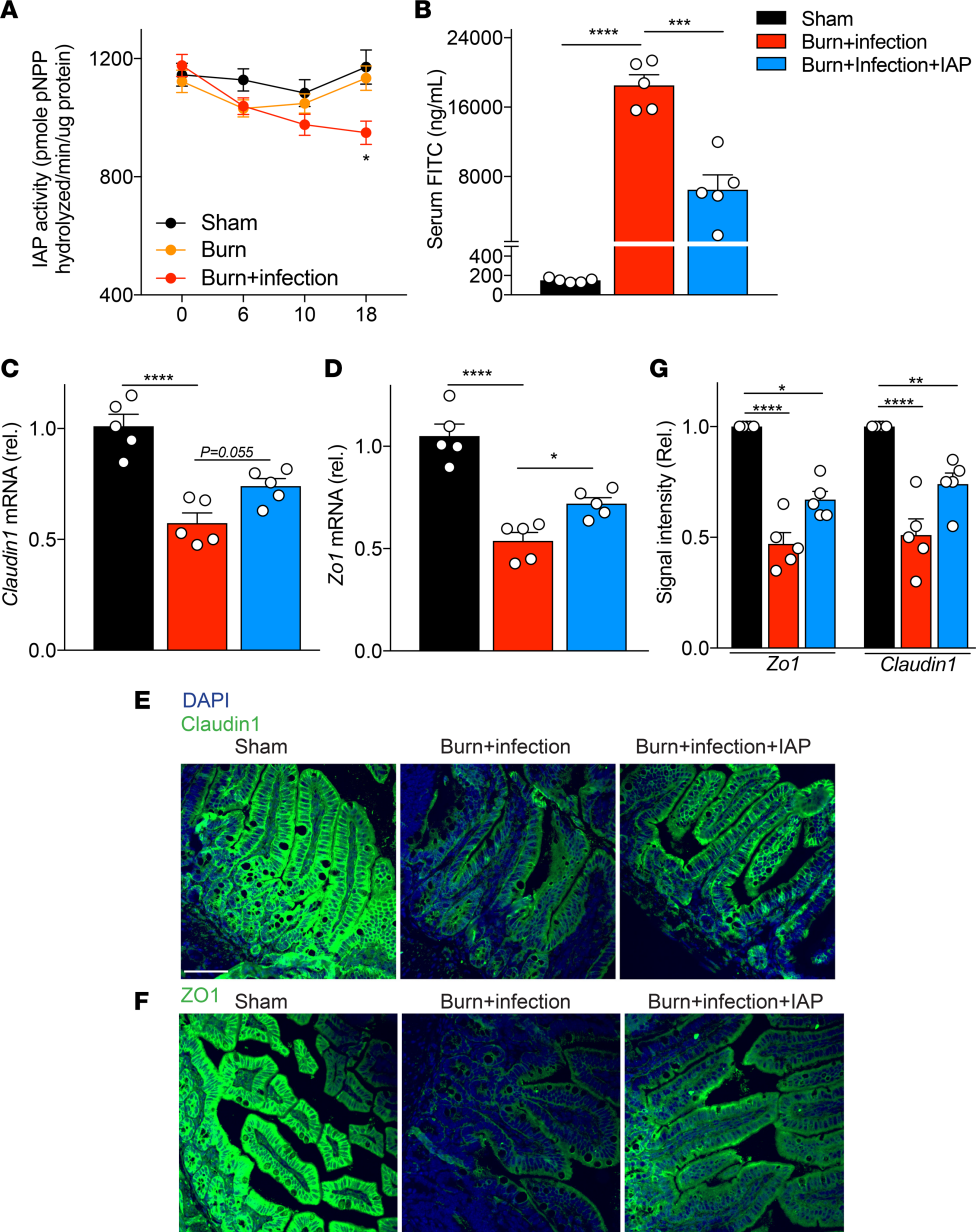
IAP supplementation reduces burn site infection–induced gut barrier damage after burn injury. (**A**) IAP activity in the stool of indicated mice measured by pNPP assay. (**B**) FITC levels at 18 hours after burn wound infection in the serum of WT animals that underwent sham procedure (black bar), burn wound infection treated with vehicle (red bar), or burn wound infection treated with IAP (blue bar). Sham group underwent a sham procedure including all the interventions except for the thermal injury. (**C** and **D**) *Claudin1* and *Zonula Occludes* 1 (*ZO1*) mRNA expression in terminal ileum at 18 hours after burn wound infection measured by qPCR. (**E** and **F**) Representative confocal microscopy images of distal ileum for Claudin1 and ZO1 ZO-1 at 18 hours after burn wound infection injury. Blue and green colors stain for DAPI or Claudin1/ZO1, respectively.) Scale bar: 50 μm. (**G**) Quantification of confocal images. One-way ANOVA with multiple post hoc comparisons using Tukey’s test was performed. Each group included 5 animals and data are representative of 3 biological replicates. *P < 0.05, **P < 0.01, ***P < 0.001, ****P < 0.0001. IAP, intestinal alkaline phosphatase; CFU, colony-forming unit.

To determine if IAP’s level may change after burn injury, we first tested IAP activity in stool of sham, burned, and burn wound–infected mice. We found a decline in IAP activity 18 hours after burn wound infection trauma (Figure 2A). Given this observation and findings from IAP-KO mice, we speculated that oral supplementation with IAP (orally gavaged, 2000 units/mouse at 3 and 12 hours after the burn infection insult) could be beneficial in the murine burn wound infection model. We observed that the mice supplemented with IAP had significantly improved gut barrier function after burn site infection compared with the vehicle-treated mice, as measured by serum FITC levels (Figure 2B). The mechanical epithelial barrier is a critical element of the intestinal barrier, and zonula occludens (ZO), occludin, and claudin are considered to be the most important TJPs in regard to the epithelial barrier integrity. We show that treatment with IAP preserves in vitro TJP expression and localization after exposure to LPS (15). In the present in vivo burn model, we evaluated TJP expression in the terminal ileum and found significantly reduced expression of *Zo1* and *Claudin1* after burn wound infection (Figure 2, C and D). Interestingly, IAP supplementation significantly restored the expression of *Zo1*. IAP considerably decreased the downregulation of *Claudin1*. However, it was not statistically significant (*P* = 0.055) (Figure 2, C and D). The beneficial effects of IAP were observed at both mRNA (Figure 2, C and D) and protein levels (Figure 2, E–G), as measured by qPCR and immunofluorescence microscopy, respectively.

### IAP supplementation diminished burn site infection–induced systemic inflammation and improved survival.

**Figure 3:**
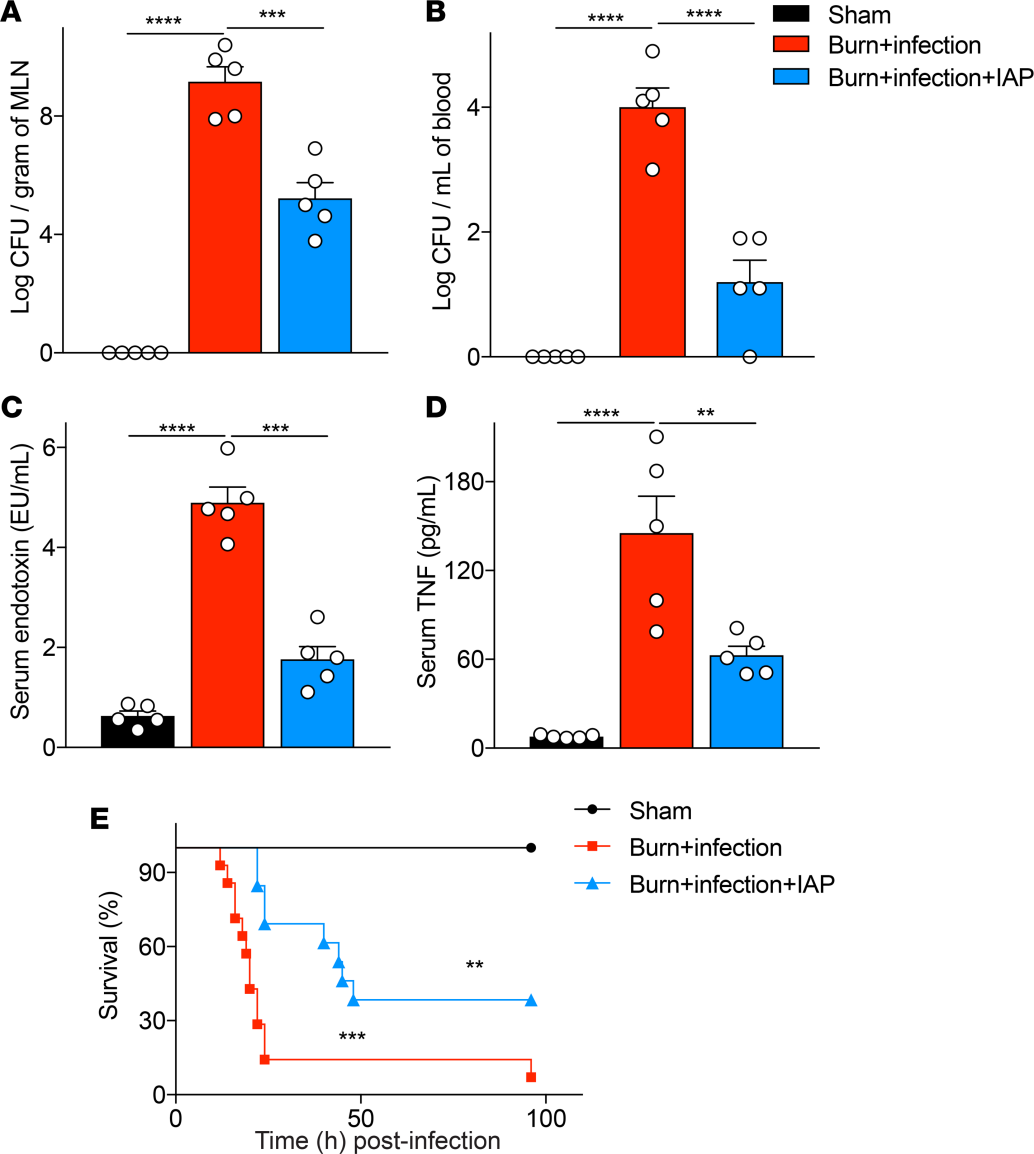
IAP supplementation diminishes burn site infection–induced systemic inflammation and improves survival. (**A**) Bacterial burden in mesenteric lymph nodes (MLNs) of mice that underwent sham procedure (black bar), burn wound infection treated with vehicle (red bar), or burn wound infection treated with IAP (blue bar), expressed as log of colony-forming units (CFUs) normalized by tissue weight. (**B**) Bacterial burden in systemic blood expressed as log of CFUs normalized by blood volume. (**C**) Serum endotoxin level measured by Limulus amebocyte lysate assay. (**D**) TNF-α levels in the serum measured by ELISA. (**E**) Survival in sham vs. burn wound–infected mice treated with vehicle or IAP. (** above the blue line denote the difference between the vehicle and the IAP supplementation group, whereas *** above the red line denote the difference between the sham and the IAP supplementation group). One-way ANOVA with multiple post hoc comparisons using Tukey’s test was performed. Kaplan-Meier survival analysis curve was used for the survival study, and the groups were compared using the log-rank test. Each group included 5 animals and data are representative of 3 biological replicates. The survival study includes 14 burn site–infected and 13 burn site–infected IAP-treated mice. **P < 0.01, ***P < 0.001, ****P < 0.0001. IAP, intestinal alkaline phosphatase.

As mentioned previously, burn-induced gut barrier damage is associated with increased bacterial translocation to MLNs and systemic inflammation, which may lead to MOF and death (23, 24). Accordingly, we confirmed our previous finding (11), that burn site infection with *P*. *aeruginosa* lead to a dramatic increase in the bacterial load in both MLNs and systemic blood and also showed that mice supplemented with IAP exhibited a significantly reduced bacterial load in both MLNs and systemic circulation (Figure 3, A and B). In corroboration, IAP supplementation significantly reduced systemic endotoxin levels and inflammation after burn site infection injury, as measured by serum LPS and TNF-α levels, respectively (Figure 3, C and D). Finally, IAP supplementation considerably increased the survival of burnt and infected mice (median survival: 20 vs. 45 hours, burn infection injury with vehicle or IAP supplementation respectively, *P* < 0.01) (Figure 3E).

### IAP attenuated liver inflammation and reduced the proinflammatory characteristics of portal serum after burn wound infection insult.

**Figure 4:**
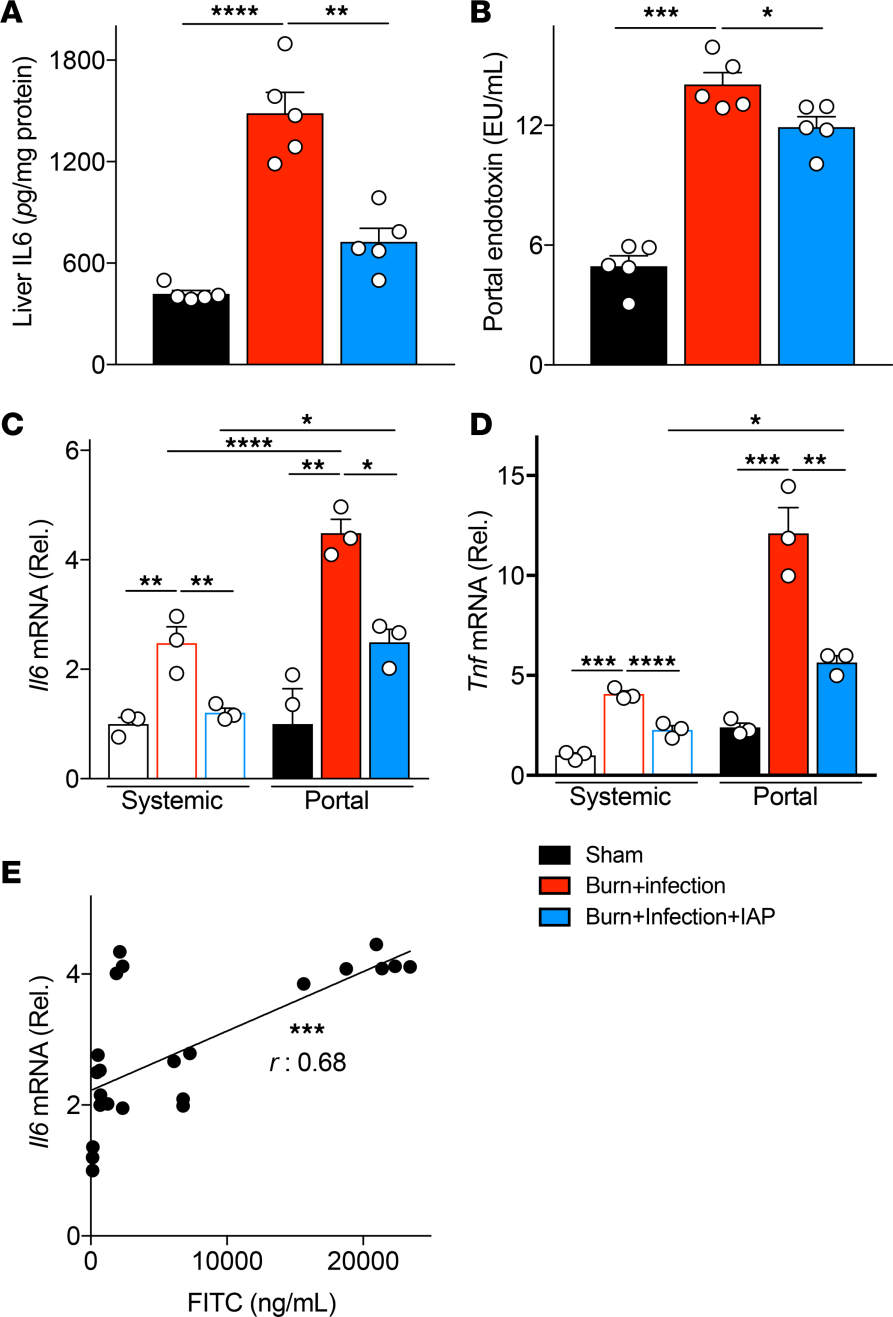
IAP attenuates liver inflammation and reduces the proinflammatory characteristics of portal serum after burn wound infection injury. (**A**) IL-6 levels in the liver of sham, burn wound–infected mice with or without IAP supplementation measured by ELISA. (**B**) Portal serum endotoxin levels measured by Limulus amebocyte lysate assay. (**C** and **D**) *Tnfa* and *Il6* mRNA levels of primary mouse BMDMs incubated with systemic or portal serum of the designated groups for 24 hours as measured by qPCR. (**E**) Correlation between the gut permeability measured by FITC and the proinflammatory characteristics of portal serum measured by *Il6* mRNA level in BMDMs. One-way ANOVA with multiple post hoc comparisons using Tukey’s test was performed for Figure 4, A–D. Pearson’s correlation coefficient was used in Figure 4E. Each group included 3–5 animals and data are representative of 3 biological replicates. *P < 0.05, **P < 0.01, ***P < 0.001, ****P < 0.0001. IAP, intestinal alkaline phosphatase; BMDMs, bone marrow–derived macrophages.

The anatomical link between the gut and systemic organs is provided by both the portal venous and the mesenteric lymphatic systems. The portal vein facilitates the gut-liver crosstalk as gut luminal inflammatory mediators travel to the liver and induce inflammation (25). These mediators can be host-derived inflammatory triggers and/or microbial-derived LPS and other PAMPS. Therefore, we next looked at the gut-liver axis contribution to the burn wound infection–induced inflammatory state. We specifically tested the proinflammatory characteristics of the portal serum after burn wound infection. As previously described (see above), IAP functions primarily within the lumen, so we hypothesized that IAP supplementation may modulate the portal vein proinflammatory effects through blocking the inflammatory triggers inside the intestinal lumen and preventing these triggers from translocating into the portal circulation. First, we looked at liver inflammation after burn wound infection injury. We found that burn wound infection significantly increases liver inflammation and that this was ameliorated with IAP supplementation (Figure 4A). LPS is one of the main bacterially derived inflammatory mediators. Given that IAP can function to block LPS, we measured the LPS level in the portal serum. Similar to our observation in the systemic serum, the amount of portal endotoxin increased dramatically after burn wound infection. However, luminal IAP supplementation significantly reduced the endotoxin levels in portal serum (Figure 4B). We then looked at the inflammatory responses of primary bone marrow–derived macrophages to the systemic and portal serums from mice that were subjected to sham injury or burn wound infection with or without IAP supplementation by measuring *Tnfa* and *Il6* mRNA levels. First, we found that portal serum triggers a significantly more profound inflammatory response in macrophages compared with the systemic serum, consistent with its more proximate location relative to the gut and its luminal inflammatory mediators (Figure 4, C and D). Furthermore, significantly reduced concentrations of inflammatory cytokines in the systemic serum confirms the critical role of the liver in clearing intestinal-derived inflammatory mediators and preventing their entrance to the systemic organs (25). Second, IAP supplementation significantly reduced both systemic and portal serum proinflammatory characteristics (Figure 4, C and D). IAP’s function of blocking endotoxin in the portal and systemic serum may be one of the main mechanisms by which IAP reduces systemic inflammation in the acute burn setting. Moreover, as mentioned before, IAP neutralizes other host- or microbial-derived inflammatory mediators, such as flagellin, ATP, and CpG DNA, inside the intestinal lumen and can also positively regulate gut barrier function (15). Taken together, all of these functions of IAP can reduce the translocation of inflammatory triggers to both systemic and portal circulations. Interestingly, we found a strong correlation between gut permeability quantity (measured by FITC) and portal serum proinflammatory characteristics (measured by *Il6* response in macrophages), confirming that burn wound infection–induced gut barrier damage can lead to a systemic inflammatory response through increasing the proinflammatory mediators of the portal serum (Figure 4E). Therefore, based on our observations, a homeostatic gut-liver axis may switch to a highly inflammatory state in response to the burn/infection insult and this can be attenuated by IAP, likely through a blockade of the host- or microbial-derived inflammatory mediators inside the intestinal lumen.

### IAP reduces burn wound infection–induced intestinal inflammation and barrier damage.

**Figure 5:**
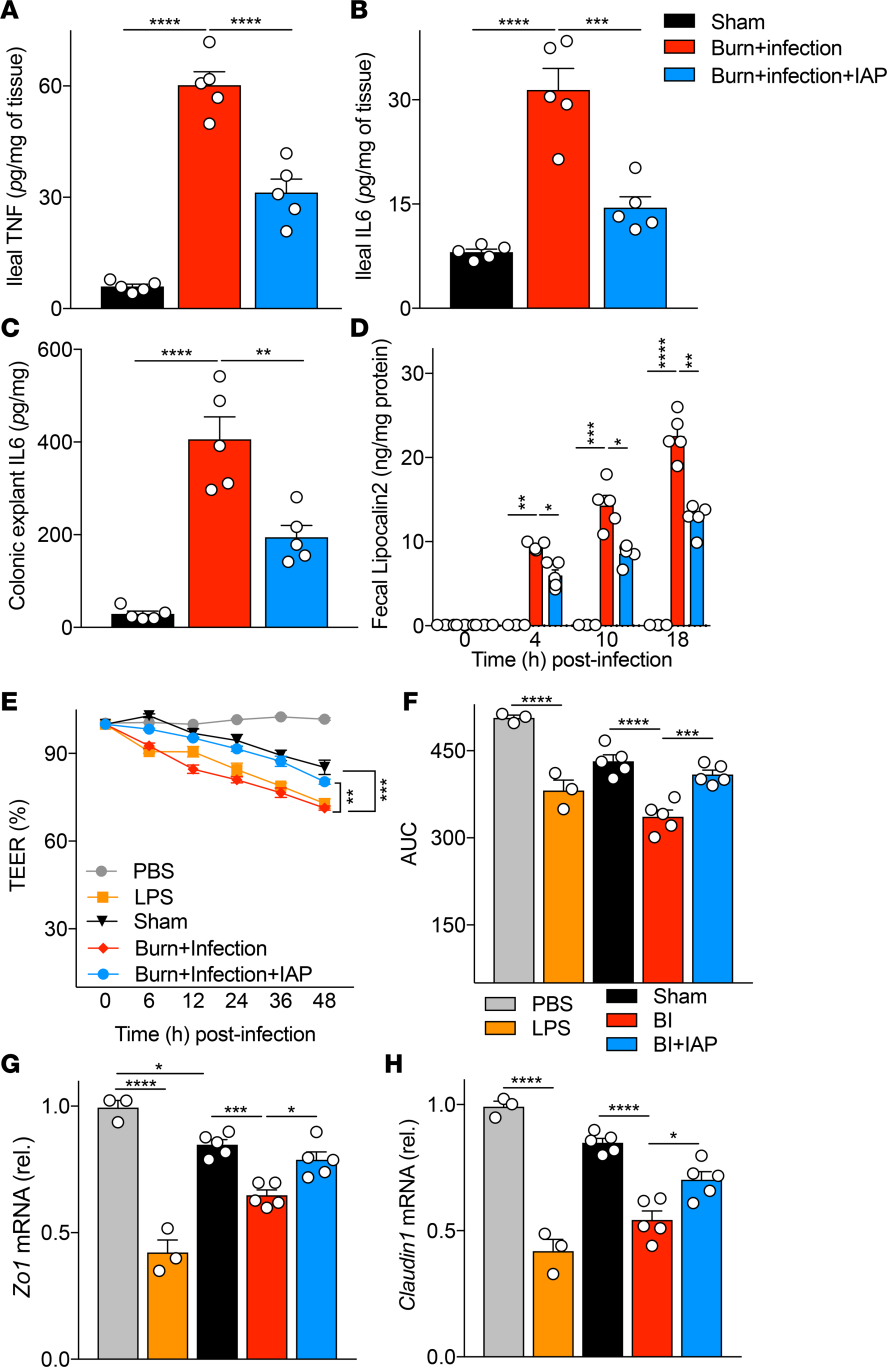
IAP reduces burn wound infection–induced intestinal inflammation and prevents the barrier damage secondary to luminal inflammatory mediators. (**A**) TNF-α and (**B**) IL-6 levels in the distal ileum tissue of sham, burn wound–infected mice with or without IAP supplementation measured by ELISA. (**C**) IL-6 levels from colonic explants measured by ELISA. (**D**) Fecal lipocalin-2 levels measured at the indicated time points and normalized by fecal weight. (**E**) Percentage of measured transepithelial electrical resistance (TEER) in Caco-2 Transwells at the indicated time points divided by measurements at the 0-hour time point. LPS (100 ng/mL) and equal amounts of PBS were used as a positive and negative control, respectively. The TEER readings were documented every 6 hours for 48 hours. (**F**) Area under the curve (AUC) values calculated for TEER readings. (**G** and **H**) *Zonula Occludes1* (*ZO1*) and *Claudin1* mRNA expression levels in Caco-2 monolayer at 48 hours after incubation with luminal contents from the indicated groups measured by qPCR. Data are expressed as mean ± SEM. One-way ANOVA with multiple post hoc comparisons using Tukey’s test was performed. Each group included 5 animals and data are representative of 3 biological replicates. *P < 0.05, **P < 0.01, ***P < 0.001, ****P < 0.0001. IAP, intestinal alkaline phosphatase.

The intestinal epithelial barrier layer separates the luminal inflammatory mediators from the host immune cells located within the lamina propria, which prevents intestinal inflammation (26). Therefore, we hypothesized that burn wound infection–induced gut barrier dysfunction can trigger a local intestinal inflammation. First, we looked at the local inflammation in the terminal ileal wall. We found significantly increased inflammation in the ileum tissue of the burn wound–infected mice with a significant reduction achieved in mice supplemented with IAP, as measured by TNF and IL-6 levels measured by ELISA (Figure 5, A and B). We also examined the colon tissue and again found greater inflammation after burn wound infection, as measured by IL-6 protein level, which was significantly reduced by IAP supplementation (Figure 5C). We also measured fecal lipocalin-2, an acute phase reactant protein that is well known to be a highly sensitive and reliable marker for intestinal inflammation (27). We found a time-dependent increase in the levels of fecal lipocalin-2 after burn wound infection insult (Figure 5D). IAP supplementation significantly reduced fecal lipocalin-2 levels, confirming the beneficial antiinflammatory effects of IAP within the intestinal lumen. It is important to note that the first dosage of IAP was administered 3 hours after the burn wound infection insult, with a subsequent reduction in the intestinal inflammation at the 4-hour time point, demonstrating that intragastric the administration of IAP immediately reduces inflammation during the clinically relevant emergency setting after burn injury.

Local intestinal inflammation affects the gut barrier function by altering the abundance of TJPs (28). Therefore, we directly looked at the capacity of the luminal contents of mice that underwent burn wound infection in triggering intestinal barrier damage using a Transwell epithelial monolayer in vitro assay, which can mimic the intestinal epithelial barrier. Interestingly, we found a dramatic barrier integrity damage with the application of luminal contents from burn wound–infected mice when compared with the application of luminal contents from control mice (Figure 5, E and F). As expected, the observed disrupted integrity was associated with downregulation of TJPs expression in the monolayer, as measured by mRNA levels of *Zo1* and *Claudin1*. (Figure 5, G and H). Furthermore, the application of luminal contents from IAP supplemented mice preserved the monolayer integrity, demonstrating that IAP treatment was able to prevent the downregulation of TJPs in the monolayer (Figure 5, E–H).

### IAP supplementation preserved gut barrier function and attenuated systemic inflammation in a cutaneous burn injury murine model.

**Figure 6:**
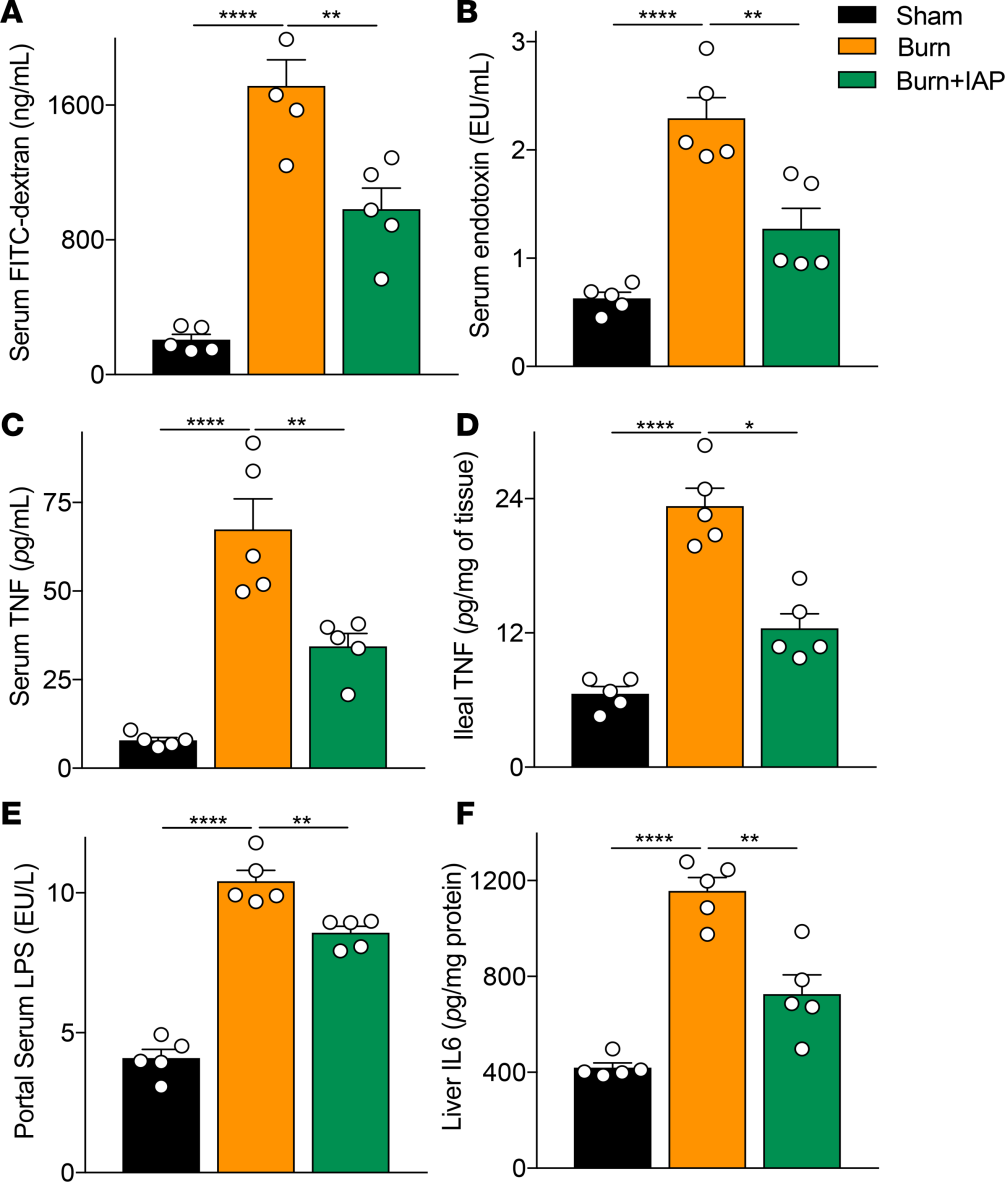
IAP supplementation preserves gut barrier function and attenuates systemic inflammation in a cutaneous burn injury murine model. (**A**) Gut permeability at 6 hours after a 30% TBSA dorsal burn insult measured by FITC-dextran levels in the serum 4 hours after intragastric FITC administration. (**B**) Serum endotoxin levels measured by Limulus amebocyte lysate assay at 6 hours after a 30% TBSA back burn insult. (**C**) TNF-α levels in the serum measured by ELISA. (**D**) Ileal inflammation measured by TNF-α levels using ELISA. (**E**) Portal serum endotoxin levels measured by LAL assay. (**F**) IL-6 levels in the liver of sham, burn alone mice supplemented with or without IAP measured by ELISA. For multiple comparisons, 1-way ANOVA with multiple post hoc Turkey’s comparisons was performed. Each group included 5 animals and data are representative of 3 biological replicates. *P < 0.05, **P < 0.01, ****P < 0.0001. IAP, intestinal alkaline phosphatase; TBSA, total body surface area.

Thermal injury itself without wound infection negatively affects gut barrier function (2). We and other groups show that in the context of a 30% total body surface area (TBSA) burn alone, intestinal permeability increases over time and peaks around 4–6 hours after the burn injury and then gradually returns to the homeostatic levels (11, 29). However, the burned mice that receive an additional eschar infection show a dramatically increased gut permeability compared with burn alone, which continues over time and leads to a greater systemic inflammation and eventual death (11). To determine whether IAP supplementation can also benefit the host after burn injury alone without wound infection, we performed a 30% TBSA dorsal burn injury murine model and supplemented the treatment group with 2000 units of IAP 2 hours after the insult. Interestingly, we found that IAP supplementation was able to significantly reduce burn-induced hyperpermeability measured by FITC levels (Figure 6A). In addition, our data suggest that burn alone triggered an intestinal and systemic inflammation (Figure 6, B–D). Notably, 30% TBSA dorsal burn injury in mice was able to negatively affect the gut-liver axis as well, with increased portal serum endotoxin levels (Figure 6E) and worsened liver inflammation, as measured by liver IL-6 level (Figure 6F). Similar to our findings with the burn wound infection model, IAP supplementation ameliorated burn-induced systemic inflammation as measured by systemic endotoxin and TNF-α levels (Figure 6, B and C). Moreover, IAP reduced the heightened TNF-α level, thus indicating decreased intestinal inflammation levels in this setting (Figure 6D). Blocking the luminal inflammatory mediators with IAP supplementation significantly reduced the endotoxin levels in portal serum and attenuated liver inflammation after the burn injury insult (Figure 6, E and F).

## Discussion

The data from the present study are consistent with previous reports that show significant gut barrier dysfunction as a result of burn injury (30–32); however, we observed even worse gut barrier dysfunction with the addition of a burn wound infection with *P*. *aeruginosa*. IAP-KO mice were more susceptible to burn site infection–induced gut barrier dysfunction, with more bacteria in their blood and lymph nodes, higher levels of serum endotoxin and TNF-α, and worsened survival compared with WT mice. IAP was expressed exclusively in the mouse proximal small intestine and functions within the lumen of the intestinal tract, suggesting that although the site of injury was remote (i.e., cutaneous burn wound infection), the gut played a major role in the ensuing inflammatory response. While it is known that IAP can prevent intestinal inflammation and barrier dysfunction in other models of gastrointestinal (GI) injury, the present findings suggest that IAP could be a potential therapeutic target for reducing burn-induced gut barrier dysfunction. This concept was confirmed in experiments on WT mice, in which supplemental enteral IAP administration after the burn injury and wound infection significantly attenuated gut barrier dysfunction, possibly by limiting the downregulation of TJP expression. Furthermore, mice supplemented with IAP had significantly less bacteria in the MLNs and blood as well as lower levels of serum LPS and TNF-α, supporting the theory that the gut is a major source of systemic inflammation in the burn wound infection model. Importantly, our experiments showed that protecting the gut barrier, in this case with enteral IAP supplementation, can significantly improve survival after burn would infection. This promising finding provides intriguing evidence to potentially support a future trial in human patients suffering from burn injury to reduce the overwhelming burden of systemic sepsis in this population. Our murine model is particularly clinically relevant because mice were administered IAP after the burn injury. Patients suffering from a thermal injury can analogously be administered enteral IAP soon after the burn insult. Furthermore, IAP is an endogenous antiinflammatory enzyme with essentially no known risk profile. Indeed, enteral IAP was shown to be of benefit in a cohort of patients with severe ulcerative colitis. Importantly, no side effects are seen in this study (33).

The canonical mechanism for IAP’s antiinflammatory effects is centered on the dephosphorylation of the lipid A moiety of LPS, resulting in monophosphorylated lipid A. The dephosphorylated LPS is a potent TLR4 agonist; however, it has negligible toxicity, if any, when compared with its precursor (34). In fact, previous studies suggest that burn-induced GI injury may be dependent on signaling through the TLR4 pathway. For example, Peterson et al. (35) found that TLR4-KO mice have less intestinal permeability to FITC-dextran than WT mice after burn injury as a result of alterations in the TJP. Furthermore, TLR4 KO mice have undetectable plasma TNF-α concentrations, unlike burned WT mice. Others show that burn-induced GI injury is dependent on IL-6 (36), a cytokine known to be upregulated with TLR4 signaling induced by infection or injury (37). In our model, IAP significantly decreased IL-6 in intestinal tissue and protected the gut barrier, possibly by decreasing LPS stimulation of the TLR4 pathway. Interestingly, expression of TLR4 in the enterocytes increases after a physiologic stressor such as burn wound infection, possibly priming the intestinal mucosa to respond to intraluminal LPS (38). It will be of interest in future studies to further explore the precise mediators and pathways that IAP works through to improve the outcomes in this burn infection model.

We show that LPS is not the only substrate for IAP dephosphorylation (13), which may give further insight into alternative mechanisms of burn wound–induced gut barrier protection. For example, Grimes et al. show that flagellin, a bacterial protein and TLR5 agonist that is also inactivated by IAP dephosphorylation, is an important regulator of burn-induced gut barrier dysfunction and dysbiosis (39), with significantly higher flagellin levels detected in the serum of burnt patients compared with healthy donor serum. Additionally, we show that IAP is able to dephosphorylate intestinal ATP and other triphosphates, thereby promoting the growth of intestinal commensal bacteria (13). Cauwels et al. found that the removal of intestinal ATP using apyrase lead to improved gut barrier in a mouse model of sepsis-induced barrier injury (40). Separate studies demonstrate that burn injury alters the intestinal microbiome composition, leading to a proinflammatory dysbiosis (41–43) that can potentially be reversed with fecal microbiota transplant (43). We show that IAP preserves the normal homeostasis of gut microbiota and promotes commensal bacterial growth by reducing the concentration of luminal nucleotide triphosphates (13). Therefore, IAP may function to prevent burn-induced gut barrier injury by dephosphorylating ATP and maintaining the commensal, barrier-protective gut microbiome populations. This theory is further supported by our data showing that enteric contents from control-treated, burn wound–infected mice are more proinflammatory and induce more barrier dysfunction in a Transwell culture model compared with enteric contents from IAP-treated mice.

Sepsis and the systemic inflammatory response are major drivers of morbidity and mortality in patients with a burn injury and occurs in a significant proportion of patients suffering from a greater than 20% TBSA burn. Despite this, there are no widely used therapies to prevent or treat this response. With a body of evidence now supporting the hypothesis that the GI tract is a motor for the development of systemic inflammation, maintenance of GI integrity becomes a potential target for improving patient outcomes. We believe this study demonstrates that maintenance of the gut barrier can improve survival in a murine model of burn wound infection. Lutmer et al. (44) demonstrated that enteral EGF prevents burn-induced gut injury and its associated multiorgan dysfunction but do not examine its impact on survival. Others demonstrate similar improvements in survival by protecting the gut in different murine models of sepsis. For example, systemic EGF decreases 7-day mortality in murine models of pneumonia and general peritonitis. Interestingly, in the pneumonia model, EGF does not improve lung pathology, but instead prevents pneumonia-induced GI mucosal injury (45, 46). Similarly, prevention of intestinal stem cell apoptosis improves survival in a model of *P*. *aeruginosa* bacteremia (47). Survival data from these studies in combination with data from our study further suggest that GI failure is important in the underlying pathogenesis of the systemic sepsis/inflammatory syndrome, and the gut should therefore be a future target of therapies in improving outcomes for critically ill patients.

While a variety of other compounds, including palmitoyl acyltransferase inhibitors (48), valproate (31), pentoxifylline (49, 50), myosin light chain kinase inhibitors (51), and nicotine (52) and procedures like vagal nerve stimulation (53, 54), are gut protective in murine models of burn-induced gut barrier injury, IAP is unique in that it is an endogenous enzyme secreted into the gut lumen by duodenal enterocytes. Furthermore, supplemental IAP is given enterally and administration is practical in a clinical setting in that it could potentially be added to the enteral nutritional formula, which is frequently initiated early in the course of burn care.

Although this study provides intriguing data that may translate to important progress in the care of patients with septic burn, it has several limitations. First, while we showed that IAP is effective in maintaining the gut barrier and improving survival, we did not determine the precise mechanism by which IAP functions in this model. This mechanism is important and its determination could spur development of additional therapies for burn/sepsis-induced gut barrier dysfunction. Second, while IAP is administered enterally and is thought to function only in the gut lumen, endogenous IAP is known to be secreted bidirectionally into the gut lumen and systemic circulation (approximately 2%–4% of IAP is thought to be absorbed systemically). The current study does not determine the potential role of systemic IAP on reducing morbidity from burn wound infection. Finally, while we observe that the gut barrier dysfunction began to decline within hours after the burn wound sepsis insult, we did not explore at which time point after injury IAP supplementation would become most effective. In this model, it was administered by gastric gavage at 2 time points, 3 and 12 hours after burn wound infection; however, it may potentially be more effective if administered continuously or in some other manner.

In summary, our study demonstrates that IAP plays a critical role in maintaining the gut barrier in the setting of burn and wound infection, and that supplemental enteral IAP administered after burn injury attenuates gut barrier injury, decreases local and systemic inflammation, and improves survival. Although it is unclear if these findings will translate to human burn patients, they generate intriguing data to support future clinical trials, especially given that IAP has already been given to humans and has minimal risks or side effects.

## Methods

### Mice.

Eight- to ten-week-old male C57BL/6/ (20–25 g) mice were purchased from The Jackson Laboratory. Specific pathogen–free IAP-KO (*Akp3^−/−^*) mice were obtained from the Burnham Institute Medical Research and bred at the Massachusetts General Hospital (MGH) animal facility to create homozygous IAP-KO, heterozygous, and WT C57BL/6 littermates. Animals were maintained in a specific pathogen–free environment at a 12-hour-light/dark cycle at MGH.

### Experimental models.

Animals were anesthetized using i.p. injection of ketamine (125 mg/kg) and xylazine (12.5 mg/kg). Then the dorsal fur was removed using an electric clipper. All animals received a subcutaneous injection of 500 μL normal saline (NS) to their back to protect from spinal cord damage during the burn injury. A 30% TBSA dorsal burn was induced by immersion in 90°C water for 8 seconds, using a 3 × 2 cm template. After the burn, animals received a 500 μL NS injection fluid resuscitation and a 100 μL subcutaneous injection of buprenorphine in NS (0.3 mg/mL) for analgesia. Sham animals underwent a sham procedure that included all the interventions except for the actual thermal injury. For burn wound infection, 100 μL of 10 mM MgSO_4_ containing 5 × 10^5^ colony-forming units (CFUs) of *P*. *aeruginosa* clinical isolate PA14 were intradermally injected at the burn eschar site immediately after the burn insult. An equal injection of 100 μL MgSO_4_ was used for the sham and the burn-alone groups. All mice were kept on heat pads and were closely monitored for complete recovery, any possible spinal injury, and water and food intake after the insult. At 3 and 12 hours after the burn wound infection insult, mice were gavaged with 2000 units of IAP (catalog P0114, Sigma-Aldrich). The same amount of vehicle (50 mM KCl, 10 mM Tris-HCl [pH 8.2], 1 mM MgCl2, 0.1 mM ZnCl2, and 50% [vol/vol] glycerol) was used as the control gavage. For the burn-alone experiments, IAP or vehicle was gavaged 2 hours after the burn insult.

### Tissue harvesting.

We have previously shown that in the context of 30% TBSA burn alone, intestinal permeability increases over time and peaks around 4–6 hours after the burn injury and then gradually returns to the homeostatic levels (11). However, the burn-injured mice that received an additional eschar infection showed a dramatically increased gut permeability compared with burn alone, which continued over time (11). Considering the difference in the timing of the gut permeability peak after burn alone versus burn wound infection, the burnt mice were sacrificed at 6 hours after the burn insult, whereas the mice in the burn site infection group were sacrificed at 18 hours after the burn wound infection insult. Blood was collected by cardiac puncture and kept on ice in the dark before centrifugation. Then, the abdomen was opened aseptically through a midline laparotomy. MLNs, ileum, colon, and ileocecal contents were aseptically harvested. Luminal contents were removed from ileum and colon samples by flushing with cold sterile PBS. The most distal stool pellets were collected as fecal samples. All samples were then snap-frozen in liquid nitrogen or stored in RNA*later* (MilliporeSigma, R0901) for better yield RNA and transferred to –80°C (catalog 74104, QIAGEN) for future assays. Stool samples were also collected at 0, 4, 10, and 18 hours after burn wound infection.

### In vivo gut permeability assay.

Intestinal permeability was evaluated by measuring the passage of FITC-dextran from the intestinal lumen to the systemic circulation. Briefly, 4 hours before euthanasia, mice were orally gavaged with 100 μL of FITC-dextran (3–5 kDa; FD4, Sigma-Aldrich) in a concentration of 440 mg/kg body weight. After 4 hours, blood samples were taken, allowed to clot for 30 minutes, and then centrifuged to obtain serum. The concentration of fluorophore in the serum was measured by fluorescent spectrophotometry (excitation: 480 nm and emission: 520 nm). Serial dilutions of FITC-dextran were used to create a standard curve according to the manufacturer’s protocol.

### Bacterial translocation.

Bacterial load in the systemic blood was assessed through serial dilution of blood in sterile PBS followed by plating on LB agar plates. MLNs were aseptically removed and homogenized in sterile PBS. The homogenized lymph nodes were serially diluted and plated on LB agar plates. All plates were incubated at 37°C and CFUs were quantified after 24 hours.

### Immunofluorescence microscopy.

The terminal ileum was fixed overnight in 4% paraformaldehyde. After antigen retrieval (Antigen Retrieval Reagent, R&D Systems), sections were blocked with normal goat serum and stained with rabbit polyclonal anti-claudin1 and anti-ZO1 (ab15098 and ab96587, respectively [Abcam]; final concentration, 1:100). Staining was performed overnight in a humid chamber at 4°C. Slices were washed and incubated with the goat anti-rabbit (ab150077 [Abcam]; RRID: AB_2630356; final concentration, 1:500; as the secondary antibody and DAPI [Abcam]).

The slices were washed and mounted for microscopy. All images were collected using a confocal microscope (Nikon A1) (×400). ImageJ (NIH) software was used for image analysis and fluorescence quantification.

### RNA extraction and quantification of gene expression by real-time PCR.

Total RNA was extracted from the tissues or cells using the RNeasy Plus Mini Kit (QIAGEN) following the manufacturer’s protocol. cDNA synthesis was performed using the iScript cDNA Synthesis Kit (Bio-Rad) following the manufacturer’s instructions. Real-time PCR was performed using the SYBR Green Master Mix (Agilent 600830, Agilent Technologies). The primers used are listed in Supplemental Table 1. RNA levels were normalized to the expression of GAPDH as a control housekeeping gene.

### ELISA.

The mouse TNF-α and IL-6 ELISA Ready-SET-Go kits (eBioscience) were used to measure the serum, liver and distal ileal TNF-α levels, and colon and liver IL-6 levels, and the mouse Lipocalin-2/NGAL DuoSet ELISA (R&D Systems) was used to measure the fecal Lcn-2 levels following the manufacturer’s instructions.

### Endotoxin quantification.

Serum LPS levels were quantified using the Limulus Amebocyte Lysate (LAL) Assay Kit (ToxinSensor Chromogenic LAL Endotoxin Assay Kit, GenScript) following the manufacturer’s instructions.

### Ex vivo colonic explants culture.

Colon tissue (~1 cm) was washed in cold PBS and then placed in a 24-well plate containing 1 mL DMEM medium with 1% antibiotics (penicillin and streptomycin) and incubated at 37°C with 5% CO_2_ overnight. IL-6 production was measured in the supernatants by ELISA according to the manufacturer’s instructions.

### In vitro monolayer Transwell model.

Human Caco-2 epithelial cells were purchased from the American Type Culture Collection and maintained in DMEM with 10% heat-inactivated FBS and 1% antibiotic–antimycotic solution obtained from GIBCO. For creating the monolayer, the Caco-2 cells were seeded onto collagen-coated polyethylene terephthalate filter supports (1-μm pore size; Corning) at a concentration of 3 × 10^5^ cells/well and were incubated in a cell culture incubator (37°C, 5% CO_2_) for up to 3 weeks. Both the apical and the basolateral side of the Transwells were fed with DMEM supplemented with 10% FBS and 1% antibiotic. After 3 weeks the integrity of the monolayer was measured by transepithelial electrical resistance measurements (TEER). When TEER peak was reached, and maintained for 3 days, the monolayer was used for subsequent experiments. PBS and LPS 100 ng/mL were used as negative and positive controls, respectively. Ileocecal contents were weighed and homogenized in PBS in the same concentrations. The homogenized ileocecal contents from each mouse were added to the growth media in the apical compartment. The cells were then returned to the cell culture incubator (37°C, 5% CO_2_) and TEER was measured every 6 hours up to 48 hours. All experiments were performed in 3 replicates for each condition. Transwell experiments were performed as previously described (15, 55). The integrity of the Caco-2 monolayer was determined by measuring the TEER of the cell monolayer using the Millicell-ERS Electrical Resistance Measuring System (Millipore) using electrodes. The electrodes were immersed in a way that the shorter electrode was in the inner well and the longer electrode was in the outer well. 200 Ω.cm2 resistance was indicated as a confluent monolayer.

### Portal serum collection.

Animals were anesthetized using i.p. injection of ketamine (125 mg/kg) and xylazine (12.5 mg/kg). They were also injected with 100 μl of 0.015 mg/mL buprenorphine for pain control. Complete anesthetization was confirmed by evaluating for a reaction to a toe pinch. A single 1-inch incision was made using a sterilized scalpel with a sterile blade into the skin on the left side of the upper abdomen, below the ribs. The peritoneum was opened using autoclaved scissors and forceps. Using a sterile cotton swab, the large and small intestines were pulled out and placed on a sterile gauze until the portal vein was visualized. The needle was inserted into the portal vein below the liver at an angle of 5°–10° to the vein, with bevel facing up. About 200 μl of portal blood was slowly taken and allowed to clot for 30 minutes and then centrifuged to obtain serum.

### Testing the proinflammatory characteristics of systemic and portal serums using mouse bone marrow–derived macrophages.

Briefly, bone marrow was flushed from tibia and femur bones of WT mice and allowed to adhere to a nontreated tissue culture plate overnight. Nonadherent cells were then differentiated to macrophages in DMEM containing 10% FBS, 1% _L_-glutamine, 1% penicillin/streptomycin, 0.1% b-mercaptoethanol, 5 ng/mL of IL-3 (PeproTech), and 5 ng/mL of Macrophage Colony Stimulating Factor (PeproTech) for 7 days. After 7 days, cells were counted and used to assess the proinflammatory characteristics of systemic and portal serum. Primary mouse bone marrow–derived macrophages were incubated in 12-well plates with systemic and portal serum of mice for 24 hours. After 24 hours, cells were washed with PBS and collected for future assays. A total of 200,000 cells were plated with 500 μL of growth media without FBS. We added 50 μL of portal or systemic serum to the growth media. *Tnfa* and *Il6* mRNA levels were measured using qPCR.

### Intestinal alkaline phosphatase assay.

The IAP assay has been previously described (21). Briefly, an individual stool sample was homogenized in water (10 mg/mL) followed by incubation on ice for 30 minutes. Thereafter, the homogenates were centrifuged twice at 4°C at 15,000*g* for 15 minutes, and the supernatants were collected to determine IAP activity as well as protein concentration. The Coomassie Blue Protein Assay (Bradford) kit from Fisher Scientific was used for protein quantification. For IAP assay, 25 μL of supernatant was mixed with 175 μL phosphatase assay reagent containing 5 mM of p-nitrophenyl phosphate (pNPP) followed by determining optical density at 405 nm. The specific activity of the enzyme is expressed as picomoles pNPP hydrolyzed/min/μg of protein.

### Statistics.

Statistical differences among groups were determined using the GraphPad Prism software. Values were compared using 1-way ANOVA, with multiple post hoc comparisons using Tukey’s test. Kaplan-Meier survival analysis curves using the log-rank test were used for the survival studies. The statistical test used is indicated in each figure legend. Results were presented as mean ± SEM. A *P* value less than 0.05 was considered statistically significant.

### Study approval.

Animal protocols were reviewed and approved by the IACUC at the MGH (protocol no. 2006N000093, 2008N0000023) and are in strict accordance with the guidelines of the Committee on Animals of the MGH, Harvard Medical School, and the regulations of the Subcommittee on Research Animal Care of the MGH and the NIH. All animals were euthanized according to the guidelines of the Animal Veterinary Medical Association.

## Author contributions

FA designed research, conducted experiments, acquired data, analyzed data, and wrote the manuscript. PC, MA, FK, MN, and VM conducted experiments, analyzed data, and wrote the manuscript. MHG conducted experiments, analyzed data, and wrote the manuscript. LGR and RAH designed research, analyzed data, and critically revised the manuscript for intellectual content. All authors revised and approved the manuscript for publication.

## Supplementary Material

Supplemental data

## Version 1 10/02/2020

Electronic publication
