# Supplementary material for: Targeting the gut to prevent sepsis from a cutaneous burn
Source: JCI Insight. 2020 Oct 2;5(19):e137128. doi: 10.1172/jci.insight.137128 (PMC7566703; doi:10.1172/jci.insight.137128)
Supplement: Supplemental data [file jciinsight-5-137128-s172.pdf]

Supporting Material

Supplementary table 1. Oligonucleotides used in this manuscript.

Primer: mouse *Tnfa*<br>Forward: 5' CCCAAAGGGATGAGAAGTT 3'<br>Reverse: 5' CTCCTCCACTTGGTGGTTTG 3'
Primer: mouse *Il6*<br>Forward: 5' TGGGAAATCGTGGAAATGAG 3'<br>Reverse: 5' CCAGTTTGGTAGCATCCATCA 3'
Primer: mouse C*laudin1*<br>Forward: 5' AGTCTTCGACTCCTTGCTGA 3'<br>Reverse: 5' ACAAAGATTGCGATCAGCCC 3'
Primer: mouse *Zo1*<br>Forward: 5' GCTAAGA GCACAGCAATGGA 3'<br>Reverse: 5' GCATGTTCAACGTTATCCAT 3'
Primer: mouse *Bactin*<br>Forward: 5' AGC GAGCATCCCCCA AAGTT 3'<br>Reverse: 5' GGGCACGGAGGC TCATCATT 3'
